# Designing Guiding Systems for Brain-Computer Interfaces

**DOI:** 10.3389/fnhum.2017.00396

**Published:** 2017-07-31

**Authors:** Nataliya Kosmyna, Anatole Lécuyer

**Affiliations:** Team Hybrid, Institut National de Recherche en Informatique et en Automatique (INRIA) Rennes, France

**Keywords:** brain computer interfaces, feedback, feedforward, guiding system, taxonomy, design space, training protocol

## Abstract

Brain–Computer Interface (BCI) community has focused the majority of its research efforts on signal processing and machine learning, mostly neglecting the human in the loop. Guiding users on how to use a BCI is crucial in order to teach them to produce stable brain patterns. In this work, we explore the instructions and feedback for BCIs in order to provide a systematic taxonomy to describe the BCI guiding systems. The purpose of our work is to give necessary clues to the researchers and designers in Human–Computer Interaction (HCI) in making the fusion between BCIs and HCI more fruitful but also to better understand the possibilities BCIs can provide to them.

## Introduction

Brain–Computer Interfaces (BCIs) allow capturing the brain activity of users by processing and classifying their brain signals with the purpose of generating commands for any computer system. There are many applications of BCIs (van Erp et al., 2012), traditionally in medicine (Wolpaw et al., 2002; interaction with locked-in patients, typing for quadriplegics) but also increasingly out-of-the lab applications such as controlling devices (e.g., car, robotic arm—Chapter 6.2 in Tan and Nijholt, 2010), user-state monitoring (e.g., workload Pike et al., 2014) for interface and task adaptation, as well as gaming (Nijholt et al., 2009).

**Figure 1:**
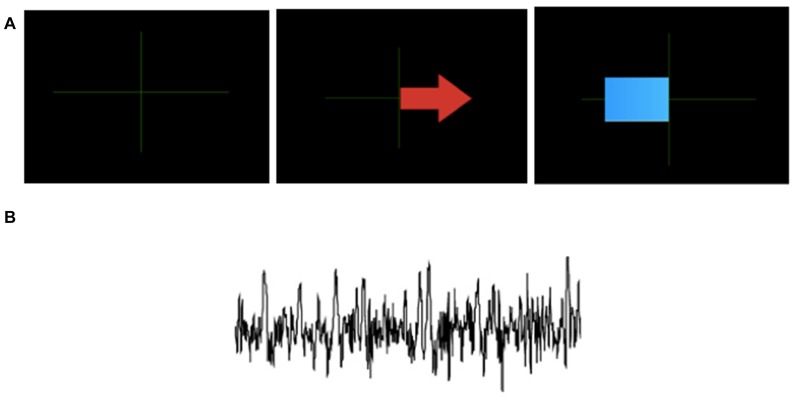
Examples of feedback in traditional brain–computer interface (BCI) training protocols (TPs; usually for expert users). Figure is reproduced with permission from the copyright holder. **(A)** Classification feedback from GRAZ BCI protocol: an arrow pointing right indicates to the learner to imagine a right hand movement. The blue bar gives feedback with its direction and length based on the classifier output. Thus it shows how well the mental task is recognized. The bar extends towards the left for an imagined left hand movement, and toward the right for the imagined right hand movement (adapted from http://OpenViBE.inria.fr). **(B)** Brain signals where only relevant activity has been preserved.

Mastering the production of stable brain signal patterns (van Erp et al., 2012) is a crucial component of all existing BCI systems. Learning this skill can be challenging, requiring hours of training and repetitive practice, especially for continuous control. Users must train to adapt their brain signal patterns to make them easy to recognize by a computer (Lotte et al., 2013). For the first BCI applications, the training technique used was Operant Conditioning (OC). OC produces good results; however, it requires extensive and painstaking training (up to several months): all the effort in learning to use the BCI is incumbent on the user. Subsequently, with the appearance of machine learning techniques, it became common to train classifiers. This allowed reduction of the training time to hours (at most), instead of days or months, by shifting the focus from the user to the system. Instead of training the user to adapt, we train a classifier to adapt to each user. However, even then, users do not have an easy way of knowing whether they imagine the action in a consistent or correct manner so that the system can recognize their brain signal patterns. One of the solutions to alleviate the severity of this issue is the use of neurofeedback (Gruzelier et al., 2006), which consists of displaying components or features of the user’s brain signals in real time (e.g., the blue bar in Figure 1A). This allows users to get a sense of how to modulate their signals during the training of a classifier, which contributes to making the job of the classifier easier. Yet, even today, feedback is designed at a low level in an often unappealing form and is thus difficult for users to understand and interpret. Currently, the improvement of feedback in BCIs is an essential step towards improving BCI performance and usability (Lotte et al., 2013). This is crucial for BCIs based on the recognition of mental imagery tasks (motor imagery (MI), visual imagery, etc.), so-called active BCIs, are the focus of this article. When a person tries an active BCI, he or she usually does not know: (1) which imagination strategies are demanded to control the application; and (2) what are the mental states associated to these commands.

There is no unified approach to address this problem, current BCI training protocols (TPs)^1^ are mostly neglecting the human and his/her needs in the loop.

In this article we propose a guiding system for feedback and feedforward mechanisms in BCIs. It should be stressed that this framework is only based on theory and its related hypotheses. As such, it is not a proven solution, and would require formal validation in the future. Nonetheless, we hope this article will provoke discussions, debates and more works on this important area of BCI research.

Our design space builds on well-known concepts in the Human–Computer Interaction (HCI) domain, such as fundamental aspects of guidance: the feedback and feedforward. From the BCI domain we inspire ourselves from design recommendations for BCIs as (Lotte et al., 2013), and part 2, chapter one from Kosmyna (2015) as there are no guiding systems and very few design options for guidance in BCIs. From the HCI domain we inspire ourselves from design recommendations from Delamare et al. (2015) initially proposed in the context of gestural interaction. We articulate and apply those recommendations to the design of BCI guiding systems. Our design space defines a unifying framework for the design of BCI guiding systems by organizing a set of design issues along axes.

## Related Work: Training in Brain–Computer Interfaces

We first review and introduce the main BCI concepts that are necessary for the understanding of this article. We will follow the steps of the “BCI loop” that symbolizes the typical BCI experience. A BCI is a closed loop between a user and the system. Generally, the user interacts with the system and the system gives feedback about its decision state after the interaction. The BCI loop is composed of (in order): the signal acquisition process; the signal processing step, in which signals are processed and prepared; the feature extraction phase, in which we identify and extract salient features from the signal; and the classification stage, in which the features are matched to the classifier model to identify one of the phenomena it was trained to capture.

The first modern BCI systems based on machine learning were trained and evaluated offline (with prerecorded signals) and were the first step towards the development of better feature extraction and signal processing techniques. Those developments then made possible the appearance of single-trial classification BCI systems that could be used online as well (i.e., used directly for a task). Among those systems, there is a distinction between *synchronous* and *asynchronous* systems.

*Synchronous* BCIs follow a two-phase protocol. First, signals from users are recorded while they perform a series of imagined actions (e.g., moving the left hand for MI-based BCIs) or while they are subjected to stimuli (e.g., flickering targets or LEDs for SSVEP BCIs). Then, a classifier is trained to recognize the different imagined actions or stimuli. Finally, the BCI system can be used online. In *synchronous* BCIs, the use of the system is not continuous (the user cannot act freely and use the system at any time). The system gives a cue to users before the stimulus is generated or before they must perform an imagined action. Then, signals are recorded for a fixed period of time (e.g., 5 s) to produce a labeled training instance. The system then gives the classification output, after which there is a resting phase (e.g., lasting 10 s) before the cycle starts over. In contrast, *asynchronous* BCIs capture and classify brain signals continuously to achieve a fully real-time system, where a user can act freely and use the system at any time (Nooh et al., 2011). As for the feedback, in synchronous systems, feedback is present during fixed periods, whereas in asynchronous systems feedback can be present all the time.

### Feedback in BCI Training Systems

In BCI systems, feedback (from the system to the user) is considered as a fundamental component of the system (Lécuyer et al., 2008) that helps users to modulate their brain activity but also to display the result of the classification. The current design of feedback for BCIs remains limited and rarely considers user satisfaction as a primary concern. As suggested by Lotte et al. (2013), there is a need for a shift in current feedback designs to get closer to practices in instructional design, in which maximizing the learning potential is key. The situation is starting to change, however, as works are appearing that use more varied or multimodal feedback.

We will first present a more detailed account of system feedback approaches, then go into possible solutions for user feedback.

#### System Feedback

System feedback is mostly concerned with showing the BCI user what the results of the recognition and classification of the brain signals are. However, the visualization of the classification output mostly remains an *ad hoc* process. There are usually few classes to choose from and the outcomes are often visualized through the resulting in-task action triggered by the BCI (e.g., when interacting with a virtual reality environment or controlling a robot). However, before real-time use, a training phase is required: a supervised training session, OC and/or a familiarization session to let the user get acquainted with the nature of BCI control. The training phase needs to be task independent and yet ground the user in the reality of the task. We can see that there is clearly a dichotomy when it comes to system feedback: on the one hand, the *task feedback*, that represents the actual performance of the user *(or just feedback in the latter of this article)*, and on the other hand, the *training feedback*, that represents the information needed to perform the task* (or feedforward in the latter of this article)*. Both terms feedback and feedforward are also explained in Section “Design Recommendations for BCI Training Protocols” of this article.

Lotte et al. (2013) summarize that BCI training feedback is often only evaluative (quantitative measure of how good the performance is) and corrective (whether the task was performed well or not). This does not match findings in instructional design and computerized formative feedback (Shute, 2008). More specifically, a common type of feedback, notably for imagery tasks, is to provide the distance from the separating plane. It is a numerical value that can then be represented by a slider or in other ways. One of the justifications for such a feedback representation is that there is an overlap with neurofeedback, in which some representations of the signals (features, frequency components) are displayed to the users in real time to help them modulate their brain signals (for a visual example see Figure 1). However, such representations imply that users must have some knowledge about the various properties of brain signals and in general how the brain and action potentials function. Acquiring such knowledge is not necessarily easy and is certainly not the most desirable requirement for the usability of the system. Furthermore, the semantics of the training phase are different from the semantics of the task itself.

In order to alleviate current limitations, one of the directions is to build BCI systems that exploit some form of user feedback.

#### User Feedback

Aside from system feedback, we have a possibility of incorporating user feedback as a way of further adapting the BCI system to the user and inducing learning. The feedback from the user to the system can either be implicit or explicit, although the latter has seldom been explored directly. Implicit feedback exploits physiological signals or activity from users to detect errors automatically (e.g., through error-related negativity (ERNs)). Explicit feedback is somewhat related to the notion of incremental training for BCI classifiers. In a traditional supervised TP, signals are first acquired offline and used to train the classifier before users can use it online. In incremental training, the classifier can be used online directly, and training examples can be added while the system is being used, which means that there could be a very short training session. Additional training examples can be captured through feedback from the user, for example. Incremental training has been shown to improve BCI performance. Millán and Mouriño (2003) demonstrated the potential of incremental training using a simple classification technique (Elmann Neural Network). They showed that the resulting classifiers after offline training and online incremental training are statistically similar. Llera et al. (2012) have proposed a theoretical model based on the idea of a “reinforcement signal” that incorporates both the notions of explicit and implicit feedback in a common framework.

### Design Recommendations for BCI Training Protocols

There are no guiding systems and very few design options for guidance in BCIs. There are few design recommendations as few options have been identified. Indeed, design options have to be identified first before studying their impact on usability.

We describe the design recommendations identified in the literature according to the two fundamental aspects of guidance: feedback and feedforward. While some studies consider both aspects, some focus only on one of them, either feedback or feedforward. We first give a brief definition of feedback and feedforward mechnisms, as these terms are mostly used in HCI, but only feedback mechanism is explicitly used by BCI community. We then discuss the guiding systems from HCI, particularly we take an example of gesture-based interaction from Delamare et al. (2015).

#### Two Categories of Axis: Feedback and Feedforward

**Figure 2:**
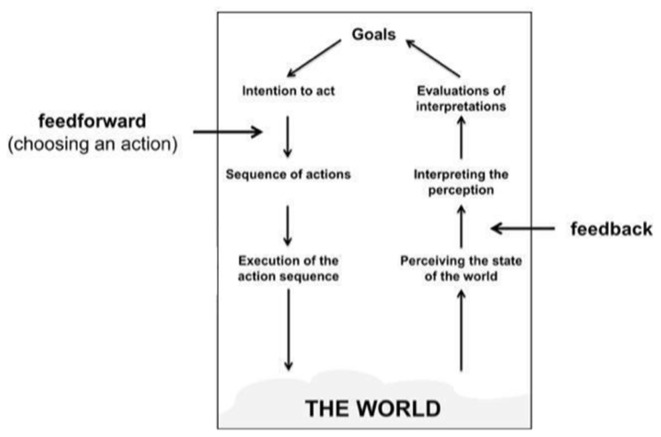
The position of feedforward and feedback in Norman’s Stages of Action model (Image adapted from Vermeulen et al., 2013).

Our design space is thus composed of two categories corresponding to two aspects for guiding systems: *feedback* and *feedforward* (Figure 2). Feedback provides information about the outcomes of the imagined actions *already performed* by the user (i.e., in the past). For a mental imagery guiding system, the feedback should provide information about the performed imagination and how well this imagination is executed or recognized (i.e., intended/recognized action). Feedforward provides information prior to any action (i.e., for future actions). For a BCI guiding system, the feedforward mechanism should contain two types of information: (a) available mental commands to the user as well as (b), how to trigger these commands using mental activity.

#### Feedback in BCIs

Lotte et al. (2013) propose three dimensions for the feedback mechanism in spontaneous BCIs.

- BCI Feedback should be meaningful. The feedback should indicate (at least roughly) when the user performed the mental task correctly. We include this dimension in our design space as “performance level” of “What” axis.
- BCI Feedback should be explanatory. The feedback should indicate the user what was performed well/wrong, and how to improve/keep this performance. Currently, BCI feedback is mostly corrective, which means that the user only obtains the result “correct/wrong” but he/she doesn’t have the clues how and why he/she managed to get this result. Please check Lotte et al. (2013) for suggestions on how to try to achieve such feedback. We include this dimension in our design space as “performance level” of “What” axis.
- BCI Feedback could be multimodal. Some research studies report using multimodal feedback, which is combined two or more modalities. Although up to now, these works have provided mixed results (a combination of audio and visual feedback decreased BCI performance Hinterberger et al., 2004), while a combination of haptic and visual feedback increased the performances (Gomez-Rodriguez et al., 2011; Ramos-Murguialday et al., 2012), we consider the multimodal feedback could be beneficial for some users and we include it in our design space, as a dimension in “How” axis.

#### Formative Feedback for BCIs

Both Lotte et al. (2013) and Kosmyna (2015) discuss the idea of formative, educational feedback for BCIs. Current BCI feedback is entirely agnostic to the many factors that can affect human learning and especially the level of mastery of the user. Depending on this level of mastery and the type of task, different strategies should be preferred in order to optimize the learning by users.

**Figure 3:**
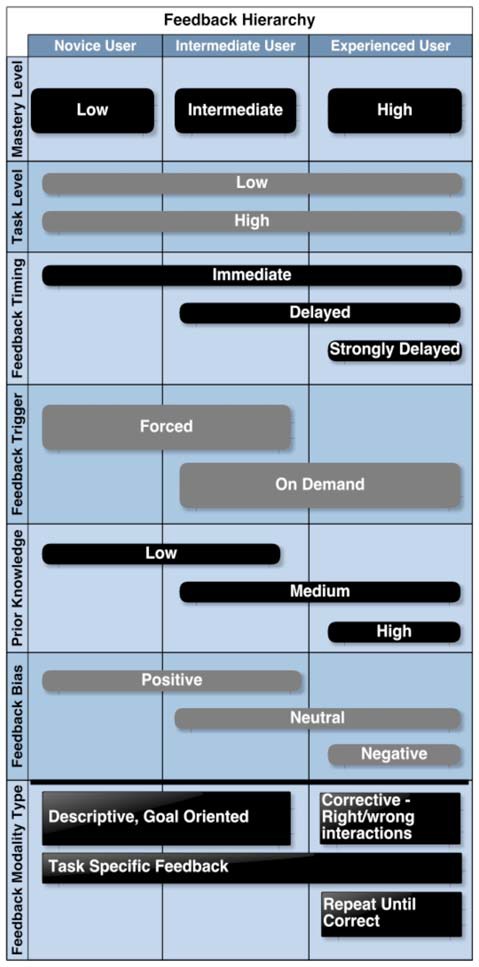
A hierarchy of feedback modalities and types for BCIs from Kosmyna (2015). Figure is reproduced with permission from the copyright holder.

If we take the example of a classroom, the individual performance of each student will strongly depend on many external factors pertaining to the learning environment and to individual successes and failures. It is to be expected that a child who is working in an environment perceived as hostile or negative and who is accustomed to failures, will not be able to reach his full potential and may be caught up in a downward spiral that completely squanders any hope of success (Shute, 2008). The study on emotional design by Plass et al. (2014) clearly identifies a strong correlation between the performance of students and the presence of positive or negative emotions. Specifically, a positive mood will favor interest and motivation, while negative emotions favor focus. In the above example where one wants to learn, positive emotions are an essential drive to learning better. When the users are in an undesirable state of mind, or have trouble understanding the objective, providing feedback can bring them to understand the situation better and guide them towards a more desirable state of mind. This type of formative feedback is typically used in educational situations where teaching is specifically tailored to one individual of a small group, as defined by Shute (2008). In education, feedback from the students to teachers is at least as essential than the converse. Indeed, such feedback provides invaluable information to the teachers about their shortcoming and allows them to improve and better tailor the content to the students. Starting from the feedback framework proposed by Mason and Bruning (2001), Kosmyna (2015) has proposed to adapt it and to make it more suitable for BCIs. She presented a feedback taxonomy, where each level corresponds to a different variable of the system that characterizes the feedback to be used as well as it various delivery modalities (Figure 3). She divided the taxonomy according to the mastery level of BCIs of the users (i.e., their experience), as feedback strategies evolve as the skill of users evolves. We will now describe in more details the framework proposed by Kosmyna (2015) in order to better position our choices for the guiding system.

#### Task Level

The nature of the task specific feedback depends on the task level. For a low level atomic task, such as moving a mouse cursor with MI, a form of very simple feedback can be the position of the cursor being updated as the users perform the action. However, for a more complex and abstract task, possibly combining several low level tasks, the nature of the feedback can take more abstract forms as well. Let us take the example of driving a radio controlled model car using MI that always goes forward and that can be turned left or right by the user. The feedback could, of course be displayed as the individual atomic feedbacks, however for the action of driving it becomes possible to overlay a higher level type of feedback, for example, recommendations on how to more easily and quickly switch between left and right in short succession.

#### Feedback Timing

Feedback timing corresponds to the time delay between the moment the user performs an interaction or a task and the time the feedback is provided to them.

This distinction relies on the fact that for novice users, skills for the use of BCIs and for the tasks in certain cases have not yet started to be established. Furthermore, users do not yet have the ability to tell how they are performing. Thus, an immediate feedback would be the most beneficial for them, than the negative effects of the interruption (Shute, 2008). On the other hand, experienced users have acquired the automatisms already and know the possible errors, their severity and how to spot them. Thus, giving immediate feedback would simply be disruptive without bringing any benefit to the users at that level. In that case, it makes sense to provide feedback only after a session so that the users can analyze it calmly.

To illustrate this more concretely, let us take the example of piano players. For a novice piano player currently practicing scales, the succession of fingers used is very important and if he or she made a mistake, the tutor would immediately intervene and correct them. Otherwise they might take bad habits that would be detrimental for later. For an advanced player rehearsing a piece of music on the other hand, an interruption mid-piece would be very detrimental and would spoil the playing session. The tutor will simply wait for the end of the piece to make remarks on some of the details.

#### Feedback Trigger

The feedback trigger type is related to the feedback delay. Indeed, forced feedback corresponds to a feedback type and timing that are imposed by the system. On the other hand, on-demand feedback corresponds to a situation where feedback will be provided only if the user asks for it.

For novice users, imposing feedback allows them to learn steadily, without adding the extra cognitive load of deciding when to get feedback. While for expert user most aspects of the interaction are automated, for novices who do not have automatisms yet, the cognitive load of the task on its own is overwhelming enough and should be prioritized. Expert users who spend very little time consciously thinking about the task itself, have more available cognitive resources as well as more experience to decide when they really need feedback.

#### Prior Knowledge

Prior knowledge corresponds to how well the user knows the task to be performed:

- Low prior knowledge means the user has never or rarely performed this task before.
- Medium prior knowledge corresponds to a user who has some experience with the task or with BCIs or with other interaction modalities.
- High prior knowledge corresponds to a user who has extensive knowledge of the task. For example for a novice BCI user with an extensive prior knowledge of the task, the task specific feedback needs to be toned down, while the BCI specific feedback has to be adapted accordingly. The same goes for a user expert with BCIs but novice with the task.

As an example, the first case corresponds to a flute player who knows a specific piece very well and who tries to play that piece on a saxophone (the task here is to play a piece on a saxophone). On the other hand the second case corresponds to a flute player who is learning how to play a new piece of music (the task here is to play a new piece on a flute).

#### Feedback Bias

According to Barbero and Grosse-Wentrup (2010), less precise and positively biased feedback can provide a positive placebo effect on BCI performance, while it is detrimental to experienced users. For more experienced users, negatively biased feedback will tend to be more effective. Indeed, in experienced users a realistic feedback is necessary that specifically points out errors committed (corrective) and that gives a somewhat harsher judgment of performance so as to motivate such users to work harder in order to improve. Other factors such as an internal vs. external incentive system have an influence on the type of feedback. Negatively biased feedback for experienced users presupposes mainly internal incentives (strong personal motivation) rather than external incentives (e.g., economical gain).

We can also make a parallel with the experiments performed by Plass et al. (2014) where it was shown that a positive affective environment benefits motivation and curiosity, which is in fact what novice users need to start learning. On the other hand an environment charged with negative emotions will mostly lead to a better focus, which is what experts users need, since they have already learned/obtained the skills needed to perform the task.

#### Feedback Modality Types

Feedback modalities correspond to the actual types of feedback that can be provided to the users either individually or jointly. Depending on the level of the user one or more types may be better adapted than others.

*Descriptive and goal oriented feedback* gives to users a series of instructions that will lead them to a correct interaction. The instructions can be displayed either as text, speech, etc. This type of feedback is ideal for novices and intermediary users who are not entirely familiar with the goal to accomplish.

*Task specific feedback*, as the name indicates, corresponds to feedback about the task itself. This type of feedback is dependent on the task and usually essential as it gives an idea of the effect of performing actions relative to the task. For example in the case of rotating objects with left and right hand MI, the task specific feedback would be the updated rotation of the object on screen. Without such feedback the user would be unable to tell if anything is happening at all.

*Corrective feedback* points out to user where and when they made mistakes and a likely explanation as to what happened. Experienced users can make the most of this type of feedback as they can effectively make use of the recommendation to rectify the error on their own.

*Repeat until correct* is a feedback type that goes in hand with corrective feedback and that makes sure the users have successfully learned how to correct their mistakes (by having them redo the action until it is successful). Such a repetition could be counterproductive and frustrating for novice users who lack most of the base knowledge.

For our guiding system we decided not to keep the “prior knowledge” axes but we reused the “feedback trigger” in our “Trigger” axis for both feedback and feedforward mechanism. We also used the “corrective feedback” and “descriptive and goal oriented feedback” notions for our guiding system.

#### Feedforward in BCIs

There are very few works on feedforward in BCIs. Lotte et al. (2013) discuss the feedforward mechanism in asynchronous BCIs. First of all, it should be mentioned that in BCIs the common term used for feedforward is *training instruction*. In current BCI training procedures, these instructions are not considered systematically, and often are omitted in the research reports. Most of the time they consist in asking the subject to perform the targeted mental task before actual BCI session, e.g., to imagine a left-hand movement, or to perform an actual left-hand movement to make sure the participants understand the task (Neuper et al., 2005). Demonstrating the skill, and/or providing the pre-training and explanations related to the task are the crucial part of any training as pointed out by instructional design literature, not only in BCIs (Hattie and Timperley, 2007; Merrill, 2007; Moreno and Mayer, 2007). This suggests, the during BCI session, the participants could be asked to remember a situation in which they may have used the task they will mentally imagine later. For example, the participants could be instructed to remember a situation in which they performed a given left-hand movement (e.g., during a sport session) before imagining it. This recall of a prior experience is expected to facilitate the learning process (Merrill, 2007), and it was shown that this experience is indeed correlated to BCI performance (Halder et al., 2011). Similarly, showing the user a demonstration of a successfully working BCI system might also increase the learning of the BCI skill (Merrill, 2007).

Based on these remarks we suggest the “What” axis of our design space on feedforward, where the imagined actions could be displayed to a user.

#### Feedback in HCI: Example of Guiding System for Gesture-Based Interaction

The world of HCI proposes hundreds of different design spaces, but we focus in this article on the example of guiding systems for gestures. As Delamare et al. (2015) argue, 2D- and 3D-based gesture systems are not yet fully present in our everyday lives because “they are not self-revealing”, e.g., users do not know when and which sets of gestures are available for the current system that they are using and for which commands, but also how to trigger them. These systems draw a parallel with BCI systems that are also not self-revealing.

Delamare et al. (2015) define several dimensions for guiding systems for gesture-based interaction through the questions “What, When, How, Where”, three of which we reuse within our design space. They present the following axes: the recognition value, filtering, update rate and perspectives, which we describe in more details in section “General Structure of the Design Space”. Our design space integrates the aforementioned axes, as well as the previously cited works from “Related Work” Section. We unify them into a common structure and extend them in order to provide a specific design space for guiding systems in BCIs.

## Our Design Space for Guiding Systems in BCIS

We start by presenting the structure of our design space—two types and three orthogonal groups. We then describe each axis of our design space.

### General Structure of the Design Space

**Figure 4:**
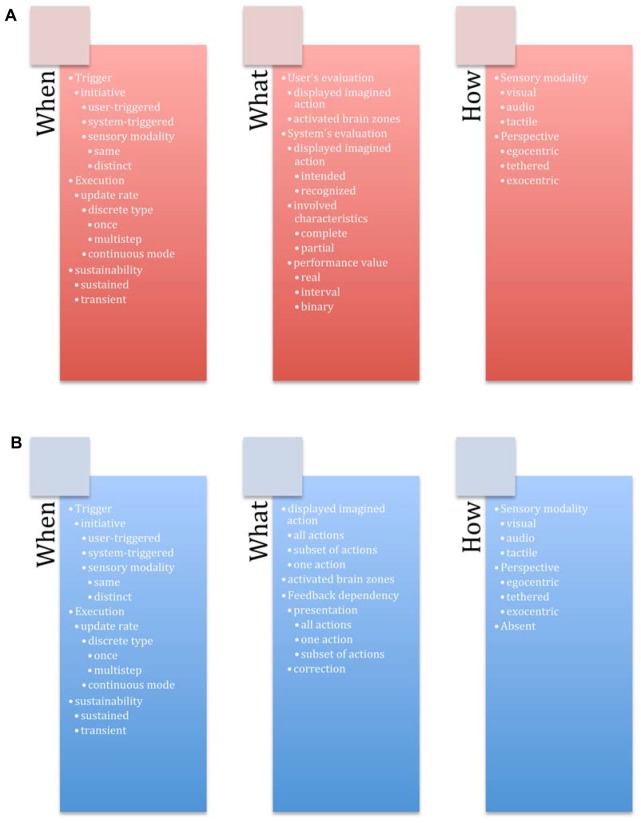
Guiding system for BCIs with 28 axes organized according to two categories—**(A)** Feedback and **(B)** Feedforward. Each category is composed of three groups—*When* (temporal characteristics), *What* (content characteristics), *How* (medium characteristics).

Our design space contains two categories corresponding to two aspects for guiding systems: *feedback* and *feedforward*. Both of these mechanisms have three groups of design axes related to three questions: *when* (temporal characteristics), *What* (content characteristics), *How* (medium characteristics). Twenty-eight axes compose our design space (Figure 4).

### Group When

This group of axes characterizes the temporal features of the feedback and feedforward mechanisms. Here we distinguish the beginning (i.e., the *trigger*), the middle (i.e., the *execution*) and the end (i.e., the end of the guiding *mode*) of the imagined action.

#### Trigger

We propose two axes for the trigger event:

- *Initiative*: the mechanism could either rely on *user only* or it could be executed by a system. For example, a *user-triggered* feedforward mechanism may display the available imagined actions if the user already used a particular mental command. A *system-triggered* mechanism could display more feedforward information if the user has difficulties imagining the actions (not recognized by the classifier, etc.).

If the choice to trigger the feedback/feedfroward mechanism was made by user, we further distinguish an axis called *sensory modality*, which defines if the user used the same type of BCI paradigm to activate the feedback/feedforward or he/she used another type of BCI paradigm, or a different input modality. The BCI systems that use more than one modality (BCI or other type) are also known in BCI terminology as *hybrid* systems. In the past few years, it has been proposed to combine BCIs with a keyboard (Nijholt and Tan, 2008), a computer mouse (Mercier-Ganady et al., 2013), or a joystick (Leeb et al., 2013). Several types of BCIs can also be used at the same time (Li et al., 2010; Fruitet et al., 2011). In Gürkök et al. (2011), participants can switch at will between a SSVEP-based BCI and a speech recognition system. Several works propose to combine BCI and gaze-tracking, for instance, using P300 (Choi et al., 2013) and MI (Zander et al., 2010) BCIs. All of the aforementioned examples are not used for feedback/feedforward mechanisms though.

#### Execution

Once the feedback or feedforward mechanism were activated, the guiding system will be executed in the following manner:

- *Update Rate*: the information can be updated in *continuous* or *discrete* way. An example of a *continuous* update rate is a feedback mechanism showing the features of the classifier in real time. It allows the user to evaluate his/her imagined actions. Another example is a feedforward mechanism providing visual representations of the possible imagined actions (for example, an image o the movement in case of MI) updated during the execution of the imagined action. A *discrete* update rate can be further described regarding its type:
- *Discrete Type*: the system can display the information *once* or *many* times. The system can display a final feedback at the end of the execution of imagined action, what illustrates the *once* option. The system can update the available mental commands each time the user performs one (for example, in the case of the drone piloting task, a system can show “Turn left”, “Turn right”, “Land” options once the user performed an imagined action to take the drone off), what illustrates a *multi-step* option. In the case of feedback mechanism, *multi-step* can also provide a possibility to the system to show more information than just a classification output (for example, it might also provide the features of the classifier, etc.).

#### Sustainability

In order to end the feedback/feedforward mechanism, we propose the following axis:

- *Sustainability*: the mechanism can be *transient* or *sustained*. Transient mechanism implies that feedback and/or feedforward will automatically disappear once the system recognizes a mental command from the user. Moreover if the system monitors the performance of the user, the system can judge that feedback and/or feedforward is no longer needed. For the case of a sustained mode, a user should select a dedicated command “close/end”. This option could be potentially interesting as if we look through the articles about BCI systems, this possibility is never discussed in feedback mechanism, e.g., feedback is either present all the time or it is absent, and the user has no control over its sustainability.

### Group What

This group of axes defines the information conveyed by the feedback/feedforward mechanisms. Feedforward mechanism provides information about the imagined actions, that will be undertaken by the user, and the feedback mechanism provides the information about past imagined actions performed by the user.

#### Group What: Mental Imagery

A guiding system provides information about: the performed imagined activity (*user’s Evaluation*), the recognized or intended imagined activity (*system’s Evaluation*), and the imagined actions managed by the system (*feedforward mechanism*).

- *User’s Evaluation*: in order to allow the user to evaluate her/his imagined activity, the system can display the *performed* (recognized by the system) imagined action or *display* the brain activation zones so the user can judge if his brain activated zones are the ones required for this task (also known as neurofeedback).
- *System’s Evaluation*: the system can present the *intended* and/or the *recognized* imagined action. The user can correct his/her imagined action by comparing the executed and the intended imagined action and by knowing what the system actually recognizes, when he/she imagines left hand movement, for example.

*Feedforward mechanism*: the system can reveal *all* available mental commands, a *subset of them* or only *one* command. For example, in the drone piloting task, a system can show only “take off” command, as it is the first one to start using the system (*one*), or it can display the “turn left” and “turn right” commands at one time (*subset*), or to show all possible commands e.g., “take off”, “move forward”, “turn left”, “turn right”, “land” (*all*). The system can also *display* the brain activation zones needed for performing the task.

#### Group What: Performance Value for Feedback

The system might evaluate the imagined actions of the user. It can compare the performed imagined action and the intended/recognized imagined action and provide the *performance value*: a *binary* value, an *interval* or a *real* value of the user performance. A binary value can be the result of a classifier such as “recognized” or “not”. A real value can be the classifier’s score for a given imagined action or a custom metric such as the distance between the intended and current imagined actions.

#### Group What: Impact of Feedback onto Feedforward

The feedforward mechanism could be impacted by the feedback mechanism, e.g., performance of the user, etc. Past actions can have two impacts on the informational content provided by the feedforward mechanism:

- *Presentation*: based on the already executed/recognized imagined action, the guiding system can remove mental actions from the initial set of mental actions available at the beginning. For example, if the performance values of the user for turning the drone left and right are very low (based on some predefined threshold), then the system can only display “moving forward”, “land”, and propose the try the “turn left”/“turn right” commands later.
- *Correction*: the guiding system can modify the content of the feedforward based on the already executed imagined actions, e.g., add or delete the information related to the already performed action, and so on.

### Group *How*

This group of axes describes how the feedback or feedforward mechanism provides guiding information:

- *Sensory Modality*: the system can transmit information through a *visual, haptic* or *audio* sensory modality. For example, it can visually display the shape of the imagined actions, use tactile stimuli to convey the scale of the deviation between intended and performed imagined actions or use a sound to indicate the success or failure of a process of imagination. Regarding the* visual* modality, as the most commonly used one nowadays, as will be discussed later, we further distinguish the *perspective*: *egocentric* (i.e., the information is presented from the user’s point of view), *exocentric* (i.e., from a third person’s point of view) or *tethered* perspective, that is attached to the user, but still separated from him/her, such as an augmented mirror with a head-coupled mechanism.

## Design Space Validation and Usage Discussion

A design space can be characterized along three dimensions (Beaudouin-Lafon, 2004):

- Descriptive power: describe a significant range of systems.
- Evaluative power: help figuring out which design options to choose.
- Generative power: help designing new systems.

**Figure 5:**
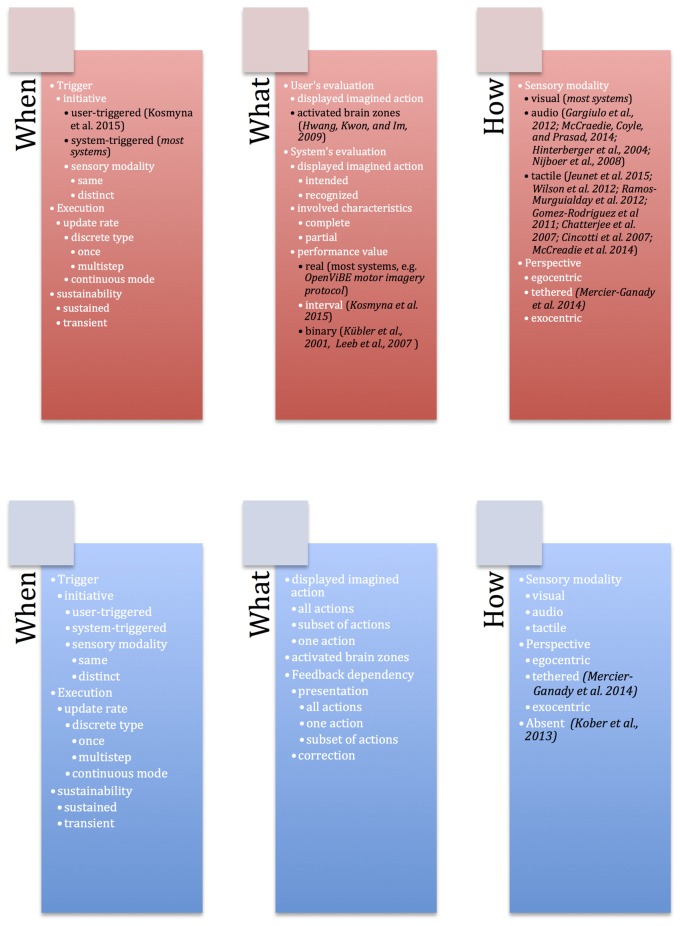
Coverage of the proposed guiding system with examples of the existing works from state of the art represented in black.

Unfortunately, as feedback and feedforward are mostly neglected aspects in BCI community, there are very few studies available so as to properly show the descriptive and evaluative power of the proposed guiding system. To assess the coverage of the guiding system, we give and discuss hereafter 10 examples of existing BCI systems (Figure 5).

We briefly explain each of the systems depending on which axes it represents.

### Feedback Axes

#### Binary Value

Kübler et al. (2001) proposed a system that displays a smiley after each successful trial. (Leeb et al., 2007) replaced the cursor with a gray smiley that moves towards the left or the right depending on the classifier output. After each trial, the smiley becomes green and happy if the trial is successful, sad and red if not.

#### Real Value

Most of the systems provide the real value, where feedback is introduced in the form of a bar or a cursor shown on screen, whose direction indicates the mental task recognized by the classifier and whose size is proportional to the confidence of the classifier in the recognized task (Pfurtscheller and Neuper, 2001).

#### Interval

Kosmyna et al. (2015) propose the visualization of the distance features of the classifier, where the user sees the distance between obtained/desired action.

#### Auditory Feedback

The classifier output in auditory feedback is represented by variations in the frequency of the sound (Gargiulo et al., 2012), its volume (McCreadie et al., 2014), or tone (Hinterberger et al., 2004; Nijboer et al., 2008).

#### Tactile Feedback

Tactile feedback has been notably used in a medical context. Wilson et al. (2012) proposed lingual electro-tactile stimulation, while Gomez-Rodriguez et al. (2011) and Ramos-Murguialday et al. (2012) focused on proprioceptive feedback (i.e., information about the limbs’ position and about the strength developed while performing a movement) and show that proprioceptive feedback allows increasing BCI performance, indicating that this type of feedback is promising for patients. Haptic feedback indicated the classifier output, either by varying the vibration patterns (e.g., different motor activation rhythms according to the classifier output; Chatterjee et al., 2007) or varying its spatial location (Cincotti et al., 2007; McCreadie et al., 2014). Jeunet et al. (2015) proposed continuously updated tactile feedback for MI task.

#### Tethered Perspective of Providing the Information

**Figure 6:**
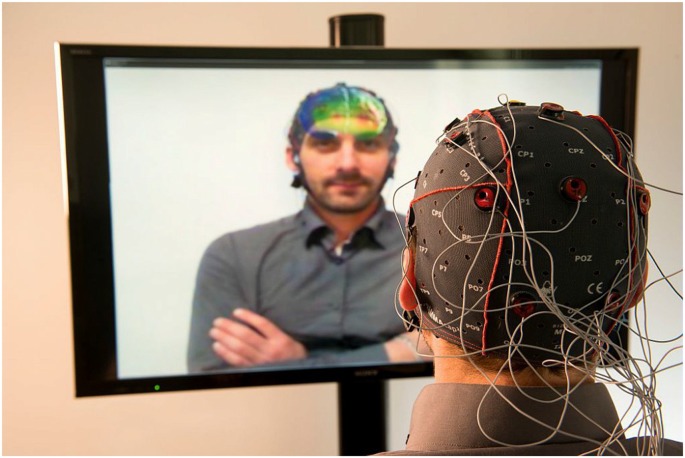
The Mind–Mirror system: a virtual brain superimposed to the real user’s image (photomontage). Figure is reproduced with permission from the copyright holders.

This setup was used for the Mind-Mirror project by Mercier-Ganady et al. (2014). It enables the visualization of the brain activity “inside of one’s head” by superimposition (Figure 6). The brain activity is extracted in real-time and is displayed in a mirror-based AR setup in front of the user’s skull in semi-transparency.

### Feedforward Axes

Neuper et al. (2005) attempt kinesthetic imagination of movements for their participants (i.e., to imagine performing the motion, feeling the same sensations, without actually moving) rather than simply visual imagination of the movements.

Kober et al. (2013), on the other hand, show that the users were not given any specific strategy at the beginning of the training phase.

The analysis of these works suggests that:

- the majority of the BCI systems only have very few features
- the feedback is nevertheless more represented than the feedforward
- the feedforward is barely considered within BCI systems

Although the cited articles all show promising results, they have not been yet been thoroughly explored by the BCI community. If we look at the feedback in BCIs, we see that the feedback provided to the user about his/her task performance is generally a unimodal (generally visual) feedback indicating the mental task recognized by the classifier together with the confidence in this recognition.

If we look more in depth on the feedforward in BCIs, very few studies have reported the mechanisms they use in order to show/explain a user how to control a BCI system. Yet this is an important element of the training process in BCIs, since this step help users to understand how to use the system. Often, this step consists only of a single directive indicating that the goal of the exercise is to “move the cursor/bar in the correct direction”.

We further analyze one existing system, that has a possibility to trigger feedback from a user (*Trigger initiative*) and we then propose a small game, with possible types of feedback that are not covered by the existing works.

### Analysis of an Existing System

We propose to analyze in more detail the system of Kosmyna et al. (2015). If we follow the axis of the feedback mechanism, the system has a *real-time system feedback*, but the user can also give the feedback to the system. It can take the following forms:

- Triggering an additional training to add supplemental references for each imagined action.
- Removing previously added reference signals (including those stemming from the initial calibration).

**Figure 7:**
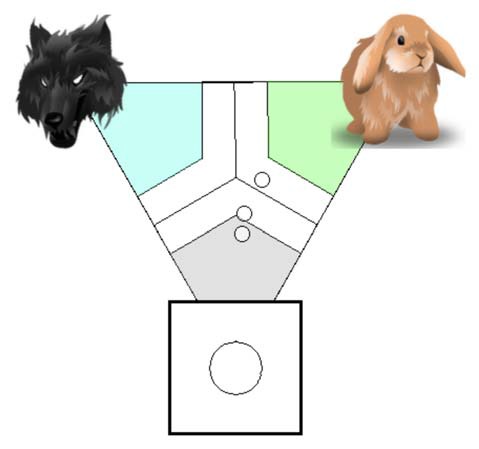
Visualization system of Kosmyna et al. (2015). Large circle that corresponds to classification outcome and several small circles that correspond to the distance n-tuples that led to that classification outcome. Figure is reproduced with permission from the copyright holder.

The feedback is introduced to a user in *visual* form. The user can close the “*user-feedback*” option but the system feedback is present at all times. The system feedback is provided in two steps: one large circle that corresponds to classification outcome and several small circles that correspond to the distances between the classes (imagination actions) that led to that classification outcome (Figure 7).

**Figure 8:**
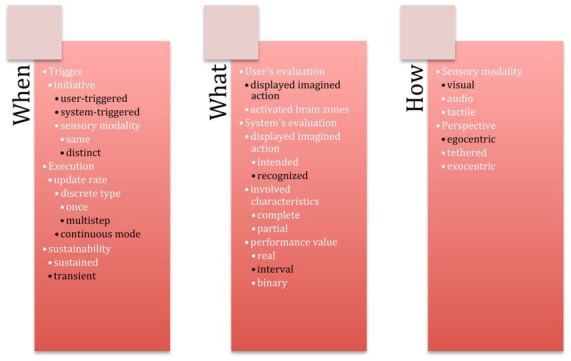
Analysis of the system of Kosmyna et al. (2015) within the proposed guiding system. The options covered by the system of Kosmyna et al. (2015) are represented in black.

The authors also explicitly represent the imagined actions by depicted images and corresponding texts. The performance level is an *interval*, which is merely represented by the proportion of distance features that are closer to each imagined action, for which the mapping in a regular polygon is done. As the authors state, “what is important is whether the points pass the classification margin or not, and near what class they are located. As for the usefulness of displaying the distance features, we believe that it can be a positive element as long as there are only a few points”. Please refer to Figure 8 to see how the system is represented within a proposed guiding system, e.g., which axes and options are implemented within the current system. For instance, as the feedback can be triggered both by the system and the user, an option *user-triggered*, as well as *system-triggered* is highlighted in black within *Trigger Initiative* axes.

As for the feedforward mechanism, the article does not provide details about it.

### Case Scenario: Helicopter Game

In order to implement and test our guiding system, we propose a preliminary application idea in the form of a game. We propose an iPad game with the following principles:

- The game involves a ship (helicopter) at the bottom of the screen, that has to collect incoming stars.
- The helicopter is always moving forward and the users may tilt the iPad left or right to make it move sideways.
- Each BCI trial corresponds to a star arriving on screen, at which moment a telescopic sight appears in the form of a crosshair.
- The star arrives from the top and moves toward the helicopter and can move from left to right and from right to left while approaching.
- The user needs to roughly align the helicopter with the star and then imagine a hand movement to catch it.
- Initially the scope shakes heavily, which makes catching the star impossible, as the user concentrates and imagines hand movement clearly (in terms of signal classification), the more the classifier feedback gets close to 1, and the less the scope shakes, thus allowing the user to catch the star.

**Figure 9:**
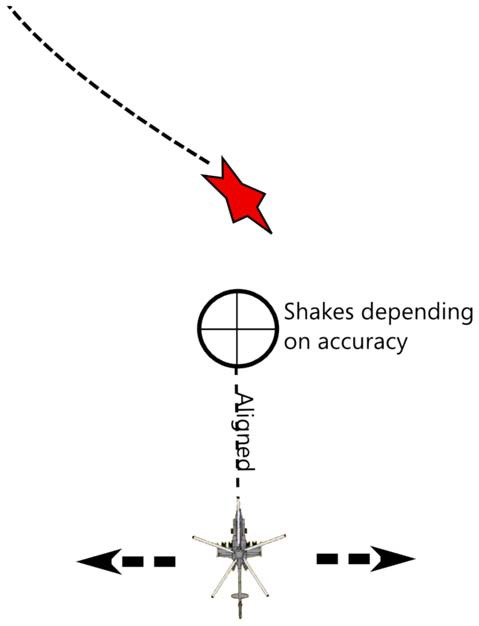
Helicopter game concept: the user needs to collect the stars while benefiting from feedback and feedforward options to learn to play the game via BCI.

Here is an illustration of what the game would look like schematically (Figure 9).

### Feedback and Feedforward Implementation in the Helicopter Game

Feedback design:

- Classification (Performance value):
  - During the “catching” phase, classification accuracy determines how much the scope of the helicopter trembles.
  - During the “catching” phase, classification accuracy is represented by the sound of the engine, a low frequency sound means a lower classification and renders the trajectory of the helicopter unstable (audio modality in our guiding system).
  - If the classification accuracy is good, encouragement text messages are displayed. If the accuracy is bad, the system displays messages to stimulate the attention of the user by proposing suggestions.
    1. If the user manages to catch a star, the game counts it as a success (system evaluation in our guiding system). The player earns a certain number of points that are added to his/her total score. The number of points added depends on the distance of the missile from the star (there is a fixed blast radius around missiles) and on the classifier.
    2. If the user fails to catch a star, a negative number of points are added to the global score (deducted) that depend on the distance of the missile from the star and of the classification correctness criterion.
    3. Ghost Mode (Feedback dependency presentation in our guiding system): another helicopter that plays perfectly is displayed in overlay; the user can see the ghost ship catch the stars and their full feedback. The objective is to push the user towards imitating this perfect play. It includes the expected position of the telescopic sight.
    4. Subjective messages: “You’re rocking it”, “Astounding”, “Marvelous”, “Not Bad”, “Careful”, “Focus!” etc. with variable level of honesty (positive bias could be introduced for the very beginners).

The ratio between the classifier performance and of the proximity of the missile to the target can be used and indicator of how much an error is due to misclassification vs. player error. The results could be provided to a user in the end of the session if he/she is interested (*user-triggered feedback* in our guiding system).

- Feedforward design:
  - Textual/video description (What axis): provide an explanation as to what the person should imagine, and how it should feel in practice (e.g., Start as though you are going to move your hand, and stop when your muscles are on the brink of contracting; do not visualize moving you hand in your head).
  - Visualization, showing the zones of the brain in specific frequency ranges, where we expect to see signals to classify.
- Game/Task Feedback transitions:
  - Imposed by the game (system-triggered): the fuel of the helicopter decreases over time, faster if the user fails to kill an enemy. When the fuel reaches zero, the resupply aircraft approaches from the side and the feedback interlude begins.
  - Chosen by the user (user-triggered): in terms of the game, the user may chose to reload the fuel supply of the helicopter if he or she feels that he or she is having difficulties controlling the game. Then, we can trigger any of the task specific feedback modalities.
  - In order to activate the feedback : an SSVEP could be used on the tablet corner; physical button press on the touch screen; shaking the iPad; tilting the iPad up, explicit BCI command, either same or distinct modality in our guiding system.
  - Imposed by the game: at the end of each level.

Add a bit of a discussion here about this case scenario…did we cover all the aspects of our design space? How well does it illustrate it? Extension possibilities?

## Conclusion

This article addresses the design of BCI guiding systems. We propose a specific guiding system that defines a framework and organizes a set of design issues along axes. The guiding system enables the study of the design of BCI guiding systems in the light of standard HCI characteristics. Our guiding system is composed of two categories corresponding to two fundamental aspects for guiding systems: *feedback* and *feedforward*. Both of these mechanisms have three groups of design axes related to three questions: *when* (temporal characteristics), *What* (content characteristics), *How* (medium characteristics). Twenty-eight axes compose our design space. The systematic experimental exploration of the proposed design space is a future direction of work. We hope that the proposed guiding system will bring the BCI and HCI community attention in order to explore the training procedures for BCIs so as to further advance the field of BCI design.

## Author Contributions

NK has prepared the manuscript, AL reviewed it and gave his feedback and useful suggestion about the improvement of the manuscript.

## Conflict of Interest Statement

The authors declare that the research was conducted in the absence of any commercial or financial relationships that could be construed as a potential conflict of interest.

## Footnotes

^1^By BCI training protocol (TP) in this article we refer to instructions and feedback only, as usually a BCI implies a training task, etc.

## References

[B1] Barbero A. Grosse-Wentrup M. (2010). Biased feedback in brain-computer interfaces. J. Neuroeng. Rehabil. 7:34. 10.1186/1743-0003-7-34 20659350 PMC2927905

[B2] Beaudouin-Lafon M. (2004). “Designing interaction, not interfaces,” in Proceedings of the Working Conference on Advanced Visual Interfaces—AVI’04 (New York, NY: ACM Press), 15–22.

[B3] Cincotti F. Kauhanen L. Aloise F. Palomäki T. Caporusso N. Jylänki P. (2007). Vibrotactile feedback for brain-computer interface operation. Comput. Intell. Neurosci. 2007:48937 10.1155/2007/48937 PMC2267023 18354734

[B4] Chatterjee A. Aggarwal V. Ramos A. Acharya S. Thakor N. (2007). A brain-computer interface with vibrotactile biofeedback for haptic information. J. Neuroeng. Rehabil. 4:40. 10.1186/1743-0003-4-40 17941986 PMC2104531

[B5] Choi J.-S. Bang J. W. Park K. R. Whang M. (2013). Enhanced perception of user intention by combining EEG and gaze-tracking for brain-computer interfaces (BCIS). Sensors 13, 3454–3472. 10.3390/s130303454 23486216 PMC3658756

[B6] Delamare W. Coutrix C. Nigay L. (2015). “Designing guiding systems for gesture-based interaction,” in Proceedings of the 7th ACM SIGCHI Symposium on Engineering Interactive Computing Systems (EICS’15) (New York, NY: ACM), 44–53.

[B8] Fruitet J. Clerc M. Papadopoulo T. (2011). Preliminary study for an offline hybrid BCI using sensorimotor rhythms and beta rebound. Int. J. Bioelectromagn. 13, 70–71.

[B9] Gargiulo G. D. Mohamed A. McEwan A. L. Bifulco P. Cesarelli M. Jin C. T.. (2012). Investigating the role of combined acoustic-visual feedback in one-dimensional synchronous brain computer interfaces, a preliminary study. Med. Devices 5, 81–88. 10.2147/MDER.S36691 23152713 PMC3496966

[B10] Gomez-Rodriguez M. Peters J. Hill J. Schölkopf B. Gharabaghi A. Grosse-Wentrup M. (2011). Closing the sensorimotor loop: haptic feedback facilitates decoding of motor imagery. J. Neural Eng. 8:036005. 10.1088/1741-2560/8/3/036005 21474878

[B11] Gruzelier J. Egner T. Vernon D. (2006). Validating the efficacy of neurofeedback for optimising performance. Prog. Brain Res. 159, 421–431. 10.1016/s0079-6123(06)59027-2 17071246

[B12] Gürkök H. Hakvoort G. Poel M. (2011). “Modality switching and performance in a thought and speech controlled computer game,” in Proceedings of the 13th International Conference on Multimodal Interfaces (Alicante: ACM), 41–48.

[B13] Halder S. Agorastos D. Veit R. Hammer E. Lee S. Varkuti B.. (2011). Neural mechanisms of brain-computer interface control. Neuroimage 55, 1779–1790. 10.1016/j.neuroimage.2011.01.021 21256234

[B14] Hattie J. Timperley H. (2007). The power of feedback. Rev. Educ. Res. 77, 81–112. 10.3102/003465430298487

[B15] Hinterberger T. Neumann N. Pham M. Kübler A. Grether A. Hofmayer N.. (2004). A multimodal brain-based feedback and communication system. Exp. Brain Res. 154, 521–526. 10.1007/s00221-003-1690-3 14648013

[B16] Jeunet C. Vi C. Spelmezan D. N’Kaoua B. Lotte F. Subramanian S. (2015). “Continuous tactile feedback for motor-imagery based brain-computer interaction in a multitasking context,” in Proceedings of the 13th International Conference on Human-Computer Interaction INTERACT (Bamberg: Springer, International Publishing), 488–505.

[B17] Kober S. E. Witte M. Ninaus M. Neuper C. Wood G. (2013). Learning to modulate one’s own brain activity: the effect of spontaneous mental strategies. Front. Hum. Neurosci. 7:695. 10.3389/fnhum.2013.00695 24151462 PMC3798979

[B18] Kosmyna N. (2015). Co-learning for Brain-Computer Interfaces. PhD Dissertation. Grenoble: Grenoble Alpes University.

[B19] Kosmyna N. Tarpin-Bernard F. Rivet B. (2015). Adding human learning in brain–computer interfaces (BCIs): towards a practical control modality. ACM Trans. Comput.-Hum. Interact. 22:12 10.1145/2723162

[B20] Kübler A. Kotchoubey B. Kaiser J. Wolpaw J. R. Birbaumer N. (2001). Brain-computer communication: unlocking the locked in. Psychol. Bull. 127, 358–375. 10.1037/0033-2909.127.3.358 11393301

[B21] Lécuyer A. Lotte F. Reilly R. B. Leeb R. Hirose M. Slater M. (2008). Brain-computer interfaces, virtual reality, and videogames. Computer 41, 66–72. 10.1109/mc.2008.410

[B22] Leeb R. Lancelle M. Kaiser V. Fellner D. W. Pfurtscheller G. (2013). Thinking penguin: multimodal brain-computer interface control of a VR game. IEEE Trans. Comput. Intell. AI Games 5, 117–128. 10.1109/tciaig.2013.2242072

[B23] Leeb R. Lee F. Keinrath C. Scherer R. Bischof H. Pfurtscheller G. (2007). Brain-Computer Communication: Motivation, aim, and impact of exploring a virtual apartment. IEEE Trans. Neural Syst. Rehabil. Eng. 15, 473–482. 10.1109/TNSRE.2007.906956 18198704

[B24] Li Y. Long J. Yu T. Yu Z. Wang C. Zhang H. (2010). “A hybrid BCI system for 2-d asynchronous cursor control,” in Engineering in Medicine and Biology Society (EMBC), 2010 Argentina: Annual International Conference of the IEEE (Buenos Aires), 4205–4208. 10.1109/IEMBS.2010.5627394 21096894

[B25] Llera A. Gómez V. Kappen H. J. (2012). Adaptive classification on brain-computer interfaces using reinforcement signals. Neural Comput. 24, 2900–2923. 10.1162/neco_a_00348 22845827

[B27] Lotte F. Larrue F. Mühl C. (2013). Flaws in current human training protocols for spontaneous brain-computer interfaces: lessons learned from instructional design. Front. Hum. Neurosci. 7:568. 10.3389/fnhum.2013.00568 24062669 PMC3775130

[B28] Mason B. J. Bruning R. (2001). Providing feedback in Computer-Based Instruction: What the research tells us. Class Research Report No. 9. Center for Instructional Innovation, University of Nebraska-Lincoln.

[B29] McCreadie K. A. Coyle D. H. Prasad G. (2014). Is Sensorimo- tor BCI performance influenced differently by mono, Stereo, or 3-D auditory feedback? IEEE Trans. Neural Syst. Rehabil. Eng. 22, 431–440. 10.1109/TNSRE.2014.2312270 24691154

[B30] Mercier-Ganady J. Loup-Escande E. George L. Busson C. Marchal M. Lécuyer A. (2013). “Can we use a brain-computer interface and manipulate a mouse at the same time?” in Proceedings of the ACM Symposium on Virtual Reality Software and Technology (Singapore), 69–72.

[B31] Mercier-Ganady J. Lotte F. Loup-Escande E. Marchal M. Lécuyer A. (2014). “The mind- mirror: see your brain in action in your head using EEG and augmented reality,” in Proceedings of the IEEE Virtual Reality (Minneapolis), 33–38.

[B32] Merrill M. (2007). “First principles of instruction: a synthesis,” in Trends and Issues in Instructional Design and Technology, 2nd Edn, eds Reiser R. A. Dempsey J. V. (Upper Saddle River, NJ: Merrill/Prentice Hall), 62–71.

[B33] Millán J. D. R. Mouriño J. (2003). Asynchronous BCI and local neural classifiers: An overview of the adaptive brain interface project. IEEE Trans. Neural Syst. Rehabil. Eng. 11, 159–161. 10.1109/TNSRE.2003.814435 12899262

[B34] Moreno R. Mayer R. (2007). Interactive multimodal learning environments. Educ. Psychol. Rev. 19, 309–326. 10.1007/s10648-007-9047-2

[B38] Nijboer F. Sellers E. W. Mellinger J. Jordan M. A. Matuz T. Furdea A.. (2008). A P300-based brain-computer interface for people with amyotrophic lateral sclerosis. Clin. Neurophysiol. 119, 1909–1916. 10.1016/j.clinph.2008.03.034 18571984 PMC2853977

[B36] Nijholt A. Reuderink B. Oude Bos D. (2009). “Turning shortcomings into challenges: brain-computer interfaces for games,” in Intelligent Technologies for Interactive Entertainment SE—15. Lecture Notes of the Institute for Computer Sciences, Social Informatics and Telecommunications Engineering, eds Nijholt A. Reidsma D. Hondorp H. (Berlin: Springer), 153–168.

[B35] Nijholt A. Tan D. F. (2008). Brain-computer interfacing for intelligent systems. IEEE Intell. Syst. 23, 72–79. 10.1109/mis.2008.41

[B37] Neuper C. Scherer R. Reiner M. Pfurtscheller G. (2005). Imagery of motor actions: differential effects of kinesthetic and visual-motor mode of imagery in single-trial EEG. Brain Res. Cogn. 25, 668–677. 10.1016/j.cogbrainres.2005.08.014 16236487

[B39] Nooh A. Yunus J. Daud S. (2011). “A review of asynchronous electroencephalogram-based brain computer interface systems,” in Proceedings of the International Conference on Biomedical Engineering and Technology (IPCBEE’11) (IACSIT Press), 11, 55–59.

[B40] Plass J. L. Heidig S. Hayward E. O. Homer B. D. Um E. (2014). Emotional design in multimedia learning: effects of shape and color on affect and learning. Learn. Instr., 29, 128–140. 10.1016/j.learninstruc.2013.02.006

[B41] Pfurtscheller G. Neuper C. (2001). “Motor imagery and direct brain-computer communication,” Proceedings of the IEEE 89, 1123–1134. 10.1109/5.939829

[B42] Pike M. F. Maior H. A. Porcheron M. Sharples S. C. Wilson M. L. (2014). “Measuring the effect of think aloud protocols on workload using fNIRS,” in Proceedings of the SIGCHI Conference on Human Factors in Computing Systems (CHI’14) (New York, NY: ACM), 3807–3816.

[B43] Ramos-Murguialday A. Schürholz M. Caggiano V. Wildgruber M. Caria A. Hammer E. M.. (2012). Proprioceptive feedback and brain computer interface (BCI) based neuroprostheses. PLoS One 7:e47048. 10.1371/journal.pone.0047048 23071707 PMC3465309

[B44] Shute V. J. (2008). Focus on formative feedback. Rev. Educ. Res. 78, 153–189. 10.3102/0034654307313795

[B45] Tan D. S. Nijholt A. (Eds.) (2010). Brain-Computer Interfaces—Applying Our Minds to Human-Computer Interaction. New York, NY: Springer.

[B46] van Erp J. B. F. Lotte F. Tangermann M. (2012). Brain-computer interfaces: Beyond medical applications. Computer 45, 26–34. 10.1109/mc.2012.107

[B47] Vermeulen J. Luyten K. van den Hoven E. Coninx K. (2013). “Crossing the bridge over Norman’s Gulf of Execution: revealing feedforward’s true identity,” in CHI’13 Proceedings of the SIGCHI Conference on Human Factors in Computing Systems (Paris, France), 1931–1940.

[B49] Wilson J. A. Walton L. M. Tyler M. Williams J. (2012). Lingual electrotactile stimulation as an alternative sensory feedback pathway for brain-computer interface applications. J. Neural Eng. 9:045007. 10.1088/1741-2560/9/4/045007 22832032

[B50] Wolpaw J. R. Birbaumer N. McFarland D. J. Pfurtscheller G. Vaughan T. M. (2002). Brain-computer interfaces for communication and control. Clin. Neurophysiol. 113, 767–791. 10.1016/S1388-2457(02)00057-3 12048038

[B51] Zander T. O. Kothe C. Jatzev S. Gaertner M. (2010). “Enhancing human-computer interaction with input from active and passive brain-computer interfaces,” in Brain-Computer Interfaces. Human-Computer Interaction Series, eds Tan D. Nijholt A. (London: Springer), 181–199.

